# Parental absence in early childhood and onset of smoking and alcohol consumption before adolescence

**DOI:** 10.1136/archdischild-2016-310444

**Published:** 2016-10-10

**Authors:** Rebecca E Lacey, Afshin Zilanawala, Elizabeth Webb, Jessica Abell, Steven Bell

**Affiliations:** Research Department of Epidemiology and Public Health, University College London, London, UK

**Keywords:** alcohol, child adversity, millennium cohort study, smoking

## Abstract

**Background:**

Parental absence, due to death or separation from a parent, has been associated with smoking and alcohol consumption in adolescence and adulthood. The aim of this study was to investigate whether parental absence in early childhood was associated with smoking and alcohol uptake before adolescence.

**Methods:**

Data on 10 940 children from the UK's Millennium Cohort Study were used. Logistic regression was used to test associations between parental absence (0–7 years) and reports of smoking and alcohol consumption at age 11.

**Results:**

Children who experienced parental absence were more likely to have smoked (OR=2.58, 95% CI 1.88 to 3.56) and consumed alcohol (OR=1.46, 95% CI 1.25 to 1.72). No differences were found by child sex or age, or parent absent. Children who experienced parental death were less likely to have drunk alcohol but those who had were more likely to have consumed enough to feel drunk.

**Conclusions:**

Parental absence was associated with early uptake of risky health behaviours in a large, nationally representative UK cohort. Children who experience parental absence should be supported in early life in order to prevent smoking and alcohol initiation.

## Introduction

Childhood adversities are known to have long-term consequences for later health, partly operating through the uptake of risky health behaviours, such as smoking and alcohol consumption.^1^ More specifically, early parental absence, has previously been shown as a risk factor for increased uptake of smoking and alcohol consumption in adolescence and adulthood.^2^ ^3^ However little is known about whether parental absence in childhood is associated with earlier uptake of these risky health behaviours prior to adolescence. Smoking and alcohol consumption initiated earlier in the life course are likely to result in the increased likelihood of dependence and reduced cessation,^4^ ^5^ which may result in increased health problems in later life.^6^

Sex differences are likely to be present in associations between parental absence and smoking and alcohol consumption. Indeed boys are more likely to initiate substance misuse in response to parental separation.^7^ The age at which childhood adversity, and in this case parental absence, is experienced is also likely to be important for risky health behaviour uptake. For instance, attachment theory suggests that disruption of the parent–child relationship in very early childhood can have lasting impacts on the child's emotional, behavioural and psychological development.^8^ It is therefore possible that parental absence occurring earlier in life might be more strongly associated with smoking and alcohol initiation before adolescence.

The aim of the present study was to provide the first evidence from a nationally representative cohort study of whether parental absence in early childhood is associated with the uptake of smoking and alcohol consumption before adolescence. We also assessed whether associations differed by child sex or age, reason for parental absence, as well as which parent was absent.

## Methods

Data from the UK's Millennium Cohort Study of 18 827 children born between September 2000 and January 2002 were used.^9^ Cohort members and their families were surveyed at the following ages: 9 months (first survey), 3, 5, 7 and 11 years. The study oversampled children from more socially deprived areas and areas with high proportions of ethnic minority families.^9^ Ethical approval was obtained from the National Health Service Research Ethics Committee and informed consent acquired from all participants.^9^

### Measures

Four parental absence variables were derived. First, parental absence was defined as change in the presence of a natural parent in the household before age 7 (binary variable: both parents present/≥1 parent absent). Second, the child's age at parental absence was categorised as: 0–9 months, 9 months–3 years, 3–5 years or 5–7 years, based on the timing of the data collection sweeps. Parental absence was captured as occurring prior to the in-home interview and reported at the time of survey. For example, a parent who was reported as absent at the 3-year survey was categorised in the 9 months–3 years window. Finally, which parent was absent (father/mother/both) and the reason for absence (death/other reason) were derived. To establish whether a change in household circumstances occurred during the cohort child's lifetime the analyses were restricted to children born to partnered mothers (n=11 716). Self-reported information on health behaviours was collected at age 11; Cohort children were asked to report whether they had ever smoked a cigarette, had an alcoholic drink or consumed enough alcohol to feel drunk. Covariates included sociodemographic characteristics as well as characteristics of the study child. These included the highest parental educational attainment (categorised as: no qualifications, overseas qualification, National Vocational Qualification (NVQ) 1, NVQ2, NVQ3, NVQ4 or NVQ5), maternal ethnicity (white, Indian, Pakistani or Bangladeshi, black or other), maternal age at birth, child's exact age at the age 11 survey, maternal smoking during pregnancy (yes or no), birth weight (kg) and gestational age (weeks).

### Statistical methods

Logistic regression was used to calculate ORs and 95% CIs for associations between parental absence and health behaviours. Model 1 estimated the crude association and model 2 included all covariates of interest (child's age, birth weight, gestational age, as well as maternal ethnicity, maternal smoking during pregnancy, maternal age at birth and educational attainment). Sex of the child–parental absence interactions were tested throughout, however none were found to be statistically significant indicating that analyses need not be sex stratified. Sex-adjusted results are therefore presented. The analytic sample consisted of singleton children living with both natural parents at birth with information on all variables of interest (n=10 940). The analyses of age at parental absence, reason for parental absence and which parent was absent were conducted on the sub-sample of children who experienced these events (n=2635). This was done by substituting these three variables into the two models in place of the binary (yes/no) parental absence variable. All analyses were weighted to account for non-response of eligible participants into the study and the unequal probability of being sampled (see above). The analyses were conducted using Stata V.13 (StataCorp. Stata V.13.1. 2013. College Station, Texas, USA. http://www.stata.com/). An α level of 0.05 was considered statistically significant.

## Results

Table 1 shows the study sample characteristics. More than one quarter of children had experienced parental absence by age 7. Few children had ever smoked by age 11, but this was more common among boys than girls. The consumption of alcohol was more common than smoking, with again more boys than girls reporting having had an alcoholic drink (14.7% vs 10.6%) or having consumed enough to feel drunk (11.9% vs 6.6%) by age 11.

**Table 1:** Characteristics of the study sample stratified by sex

	Boys (n=5460) N (%)*/mean (SD)	Girls (n=5480) N (%)*/mean (SD)	p Value for sex difference
Parental absence (0–7 years)			
No	4145 (71.4)	4160 (72.5)	0.33
Yes	1315 (28.6)	1320 (27.5)	
Age at parental absence†			
Birth to 9 months	92 (6.3)	91 (5.9)	0.30
9 months–3 years	620 (50.7)	606 (48.4)	
3–5 years	360 (26.0)	378 (29.7)	
5–7 years	243 (17.1)	245 (16.0)	
Which parent absent†			
Father	1254 (95.2)	1245 (93.2)	0.32
Mother	35 (2.6)	38 (3.9)	
Mother and father	26 (2.2)	37 (3.0)	
Reason for absence†			
Death	30 (1.8)	25 (1.4)	0.41
Other reason	1285 (98.2)	1295 (98.6)	
Ever smoked (11 years)			
No	5295 (96.4)	5390 (98.1)	<0.001
Yes	165 (3.6)	90 (1.9)	
Ever drank (11 years)			
No	4719 (85.3)	7998 (89.4)	<0.001
Yes	741 (14.7)	482 (10.6)	
Ever got drunk (11 years)‡			
No	662 (88.1)	452 (93.4)	<0.001
Yes	79 (11.9)	30 (6.6)	
Mother smoked during pregnancy			
No	4057 (71.6)	4131 (72.0)	0.74
Yes	1403 (28.4)	1349 (28.0)	
Maternal age at birth (0 years)	28.5 (5.8)	28.6 (5.8)	0.42
Highest parental education (9 months)			
None	357 (7.6)	389 (8.0)	0.99
Overseas qualification	98 (1.7)	99 (1.7)	
NVQ1	257 (5.5)	277 (5.8)	
NVQ2	1300 (26.1)	1297 (25.9)	
NVQ3	928 (16.8)	911 (16.5)	
NVQ4	2100 (35.7)	2065 (35.2)	
NVQ5	420 (6.7)	442 (6.9)	
Gestational age,§ weeks (0 years)	40.0 (38.6, 40.9)	40.0 (39.0, 40.9)	0.11
Birth weight, kg (0 years)	3.4 (0.6)	3.3 (0.6)	<0.001
Mother's ethnicity (0 years)			
White	4708 (87.7)	4665 (87.3)	0.16
Indian	155 (2.5)	139 (2.0)	
Pakistani/Bangladeshi	331 (4.5)	393 (5.5)	
Black	134 (2.7)	141 (2.6)	
Other/mixed	132 (0.3)	142 (2.6)	
Child's age at age 11 survey, years	11.2 (0.34)	11.2 (0.33)	0.27

*Weighted percentages shown.

†Of those who had experienced parental absence (n=2635).

‡Of those who had ever drunk (n=1223).

§Median and IQR reported as gestational age was negatively-skewed. NVQ, National Vocational Qualification.

Table 2 shows the results of the logistic regression analyses. Children who experienced parental absence had over 2.5-fold increased odds of having smoked before adolescence than those who were still living with both natural parents at age 7 (OR=2.58, 95% CI 1.88 to 3.56). Similarly children who experienced parental absence were more likely to have consumed alcohol by age 11 (OR=1.46, 95% CI 1.25 to 1.72). Both of these associations were robust to adjustment for confounders. Among those who had tried alcohol, children experiencing parental absence were twice as likely to have consumed enough to feel drunk (OR=2.19, 95% CI 1.32 to 3.63). Upon adjustment for covariates this association was attenuated to non-significance. The age at parental absence and whether it was the mother or father who was missing did not have statistically significant effects in associations between parental absence and smoking and alcohol consumption. Reason for parental absence was associated with alcohol consumption; children who experienced parental death were less likely to have consumed alcohol (OR=0.29, 95% CI 0.09 to 0.94), but those who did drink were more likely to consume enough to feel drunk compared with children who lost a parent for another reason (OR=12.37, 95% CI 1.56 to 98.43).

**Table 2:** ORs (95% CIs) for the association between parental absence before age 7 and smoking and alcohol consumption at age 11

	Model 1 (crude association)	Model 2 (multivariable adjusted*)
	OR (95% CI)	OR (95% CI)
*Smoked by age 11*		
All children (n=10 940)		
Parental absence		
No	Reference	Reference
Yes	2.58 (1.88 to 3.56)	1.54 (1.07 to 2.20)
Children who experienced parental absence (n=2635)		
Age at parental absence		
Birth to 9 months	Reference	Reference
9 months–3 years	1.46 (0.57 to 3.72)	1.57 (0.59 to 4.19)
3–5 years	2.03 (0.77 to 5.37)	2.64 (0.95 to 7.31)
5–7 years	1.75 (0.65 to 4.69)	2.57 (0.90 to 7.39)
Which parent absent		
Father	Reference	Reference
Mother	0.32 (0.05 to 2.06)	0.31 (0.05 to 2.14)
Mother and father	0.80 (0.17 to 3.70)	0.72 (0.16 to 3.22)
Reason for absence		
Death	0.97 (0.28 to 3.41)	1.00 (0.27 to 3.72)
Other reason	Reference	Reference
*Drank alcohol by age 11*		
All children (n=10 940)		
Parental absence		
No	Reference	Reference
Yes	1.46 (1.25 to 1.72)	1.27 (1.06 to 1.53)
Children who experienced parental absence (n=2635)		
Age at parental absence		
Birth to 9 months	Reference	Reference
9 months–3 years	1.59 (0.87 to 2.91)	1.57 (0.84 to 2.93)
3–5 years	1.38 (0.76 to 2.50)	1.39 (0.74 to 2.60)
5–7 years	1.22 (0.64 to 2.32)	1.26 (0.64 to 2.46)
Which parent absent		
Father	Reference	Reference
Mother	0.63 (0.24 to 1.66)	0.61 (0.23 to 1.58)
Mother and father	1.47 (0.57 to 3.80)	1.54 (0.59 to 4.01)
Reason for absence		
Death	0.28 (0.08 to 0.91)	0.29 (0.09 to 0.94)
Other reason	Reference	Reference
*Drunk enough to feel drunk by age 11*		
All children (n=10 940)		
Parental absence		
No	Reference	Reference
Yes	2.19 (1.32 to 3.63)	1.75 (0.99 to 3.10)
Children who experienced parental absence (n=2635)		
Age at parental absence		
Birth to 9 months	Reference	Reference
9 months–3 years	2.22 (0.57 to 8.59)	1.20 (0.30 to 4.86)
3–5 years	1.63 (0.38 to 6.94)	2.31 (0.59 to 9.07)
5–7 years	2.04 (0.42 to 9.84)	1.87 (0.44 to 7.91)
Which parent absent		
Father	Reference	Reference
Mother	0.88 (0.10 to 7.88)	1.10 (0.11 to 11.08)
Mother and father	Not estimated	Not estimated
Reason for absence		
Death	12.46 (1.62 to 96.17)	12.37 (1.56 to 98.43)
Other reason	Reference	Reference

*Adjusted for maternal age at birth, gestational age, maternal smoking during pregnancy, highest parental education level, mother's ethnicity and the child's age, birth weight and sex and.

## Discussion

Using nationally representative data from the UK we found that parental absence in early childhood was associated with increased risk of smoking and alcohol consumption before adolescence. This is supported by previous work showing that parental absence is a risk factor for adolescent and adult health behaviours, such as smoking^2^ and increased alcohol consumption.^3^ However the present study extends this previous work to suggest that differences in the uptake of risky behaviours may appear earlier in life. There is a suggestion of an increased risk of diseases, such as lung cancer and cardiovascular disease, when smoking is initiated at an earlier age.^6^ There is also a raised risk of nicotine and alcohol dependence when these substances are consumed from an early age.^4^ ^5^ Associations between parental absence and early smoking and alcohol consumption may operate through a range of mechanisms, such as reduced parental supervision, self-medication and adoption of less healthy coping mechanisms.^2^ For instance, nicotine in particular demonstrates psychoactive properties and may have benefits for mood regulation.^10^

Few previous studies have investigated sex differences with a nationally representative longitudinal sample of children, however none were found here. Our findings also suggested that the age at parental absence and whether it was the mother or father who was absent were not associated with smoking and alcohol consumption. The implications of childhood adversities for later health problems have previously been shown not to vary by age in some studies. However we did find that the reason for parental absence might have an influence on early alcohol consumption as parental death appeared to increase the amount of alcohol consumed by children who took up this risky health behaviour early in life. However given the few children who had experienced parental death and consumed alcohol in this cohort, the CIs for the association between reason for absence and alcohol are quite wide. For this reason, replication of this potentially important and novel finding in other studies is warranted.

This study is not without its limitations. As with all longitudinal studies, missing data are a potential source of bias. An investigation of our analytic sample suggested that participants with missing information were more socially disadvantaged than those included (data not shown). Hence our findings are likely to be underestimates of the true associations. We also did not include information on parental absence between 7 and 11 years, to ensure that our exposure occurred before our outcome, and it is possible that parental absence during this period may have influenced the uptake of risky health behaviours at age 11. Despite this, our study has a number of strengths. We used a large, nationally representative study of UK children with prospectively-reported information to assess the relationship between parental absence and risky health behaviours. We also had data on smoking and alcohol consumption from an early age (age 11), making this study possible.

In summary our findings suggest that parental absence is associated with increased risk of smoking and alcohol consumption prior to adolescence. Consequently early life might be an important time to intervene in order to prevent the uptake of risky health behaviours. Health behaviours established earlier in life are known to track into adulthood. Thus the early uptake of risky health behaviours is a feasible mechanism through which disparities in disease outcomes might emerge.
